# A Rare Case of Lichen Planus Mimicking Secondary Syphilis: The Great Imitator Unveiled

**DOI:** 10.7759/cureus.79845

**Published:** 2025-02-28

**Authors:** Iman Bouchelkia, Jaime Tschen

**Affiliations:** 1 Dermatology, Tilman J Fertitta Family College of Medicine, Houston, USA; 2 Dermatology, St. Joseph Dermatopathology, Houston, USA

**Keywords:** atypical lichen planus, dermatology case report, differential diagnosis, erythematous papules, skin histopathology, syphilis mimicry

## Abstract

Secondary syphilis, often referred to as "The Great Imitator," is a diagnostic challenge due to its ability to mimic a range of dermatological conditions. Accurate differentiation is critical, particularly in patients with immunocompromised states, as misdiagnosis can lead to inappropriate management. This case highlights a unique presentation of lichen planus resembling secondary syphilis.

A 68-year-old female undergoing chemotherapy for breast cancer presented with asymptomatic pink, scaly plaques on her palms and mucosal patches on her tongue. Concern for secondary syphilis prompted a serologic workup, including a rapid plasma reagin (RPR) test, which was non-reactive. Physical examination revealed no cervical, axillary, or inguinal lymphadenopathy, a hallmark feature of secondary syphilis. A 3 mm punch biopsy of the palmar lesions showed findings characteristic of lichen planus, including hyperkeratosis, hypergranulosis, and a band-like lymphocytic infiltrate. Special stains for spirochetes were negative. The patient was treated with apremilast (Otezla®) 30 mg orally twice daily and clobetasol 0.05% cream applied twice daily. After two weeks, significant improvement in the lesions was observed.

This case underscores the diagnostic complexities of lichen planus mimicking secondary syphilis. Secondary syphilis commonly presents with systemic symptoms and lymphadenopathy, which were absent in this patient. Histopathologic confirmation played a pivotal role in differentiating the two conditions. This case adds to the growing body of knowledge on atypical presentations of lichen planus in immunocompromised patients. Clinicians should maintain a broad differential diagnosis for palmoplantar and mucosal lesions. Histopathology remains a cornerstone in confirming the diagnosis and guiding appropriate management.

## Introduction

Lichen planus (LP) is a chronic inflammatory disease affecting the skin, mucous membranes, nails, and hair follicles [1]. LP is considered a T cell-mediated autoimmune disease, in which cytotoxic CD8^+^ T-cells are recruited into the skin, resulting in an interface dermatitis, characterized by inflammation at the junction between the epidermis and dermis [2-5]. Cutaneous lichen planus classically presents with red to brown, violaceous, polygonal, slightly scaling, and itchy flat papules [6]. Commonly involved sites include the wrists, lower back, ankles, and oral mucosa [7]. However, LP can present atypically, mimicking other dermatoses. Palmoplantar and mucosal involvement can pose significant diagnostic challenges, particularly in immunocompromised patients, due to its overlap with various infectious and noninfectious conditions [8].

Secondary syphilis, often referred to as "The Great Imitator," is renowned for its ability to mimic a wide range of dermatological conditions [9]. It typically follows untreated primary syphilis, which most often manifests as a solitary, painless chancre that develops at the site of infection an average of three weeks after exposure to *Treponema pallidum* [10]. Without treatment, the blood-borne spread of *T. pallidum* over the next several weeks to months results in secondary syphilis, characterized by numerous clinical manifestations. The most common features include fever, lymphadenopathy, diffuse rash, and genital or perineal condyloma lata [11,12]. Distinguishing between these two conditions is critical, as syphilis requires systemic antibiotic therapy, with parenteral penicillin as the mainstay of treatment, while LP is typically managed with topical or other immunosuppressive drugs [13,14].

This case highlights a rare presentation of lichen planus mimicking secondary syphilis in a 68-year-old immunocompromised female. The diagnostic complexity and the role of histopathological confirmation are discussed, emphasizing the importance of maintaining a broad differential diagnosis in such scenarios.

## Case presentation

**Figure 1:**
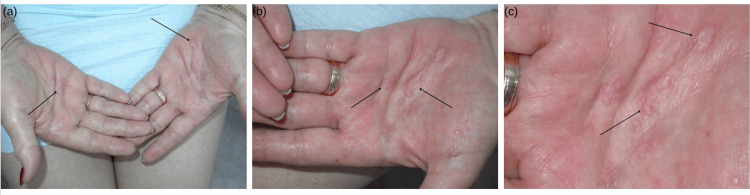
Pink, scaly plaques in a 68-year-old female undergoing chemotherapy (a) Bilateral palms with visible lesions, marked by arrows, indicate areas of discoloration and mild induration. (b) Focuses on the left palm, highlighting localized plaques indicated by arrows. (c) Close-up of the left palm, demonstrating multiple macules with erythema and scaling, as indicated by arrows.

We present the case of a 68-year-old immunocompromised female undergoing chemotherapy for breast cancer who presented with asymptomatic pink, scaly plaques on the palms (Figure 1) and mucosal patches on her tongue. Concern for secondary syphilis prompted a referral to dermatology for further evaluation. 

**Figure 2:**
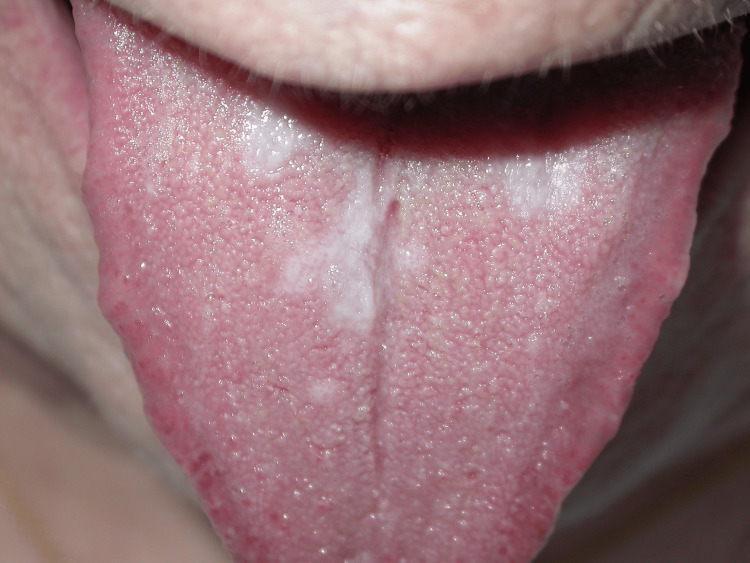
Lacy white patches on the tongue, suggestive of mucosal involvement

On physical examination, the plaques were well-demarcated with a slightly violaceous hue, but no cervical, axillary, or inguinal lymphadenopathy was noted. Oral examination revealed lacy white patches on the lateral aspects of the tongue (Figure 2). The patient denied fever, fatigue, weight loss, or other systemic symptoms.

**Figure 3:**
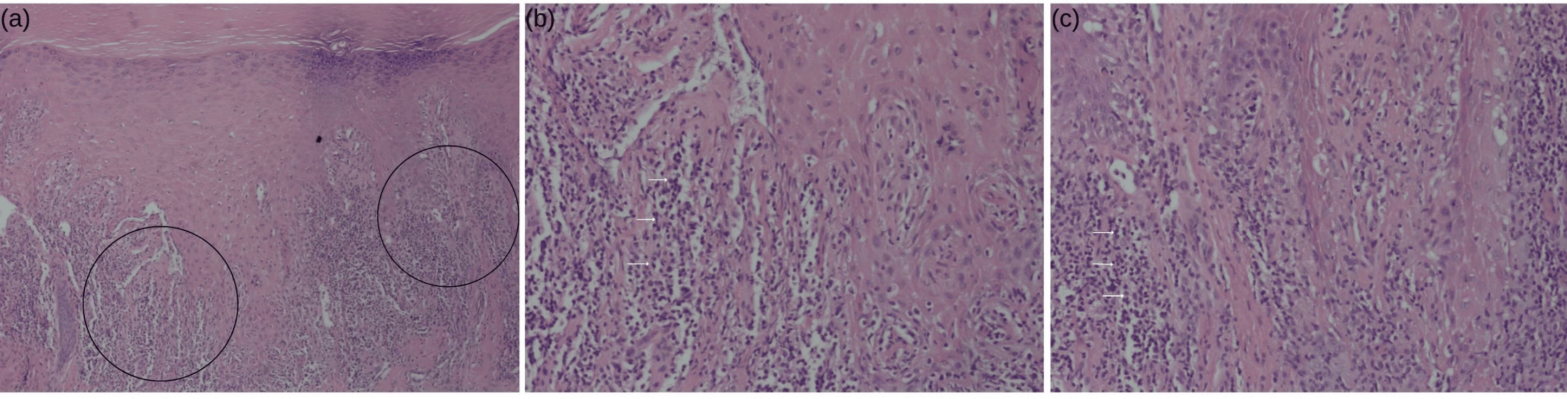
Histopathological section with H&E staining taken from a 3 mm punch biopsy of the affected skin. Images demonstrate two magnifications of the dermo-epidermal junction, highlighting findings consistent with lichen planus. (a) Low-power magnification (10x) reveals hyperkeratosis, hypergranulosis, and a dense, band-like lymphocytic infiltrate at the dermoepidermal junction; (b) Magnification (50x) from Figure 3a; (c) Magnification (50x) from Figure 3a.

A serologic workup, including a rapid plasma reagin (RPR) test, was performed and returned non-reactive. To further evaluate the palmar lesions, a 3 mm punch biopsy was taken. Histopathological analysis revealed findings characteristic of lichen planus, including hyperkeratosis, hypergranulosis, and a band-like lymphocytic infiltrate at the dermo-epidermal junction (Figure 3). Special stains for spirochetes were negative, speaking against syphilis, as further confirmed with negative RPR.

The patient was treated with apremilast (Otezla®) 30 mg orally twice daily and clobetasol 0.05% cream applied to the affected areas twice daily. Within two weeks of initiating therapy, the lesions showed significant improvement. 

## Discussion

This case emphasizes the diagnostic complexities of differentiating between lichen planus (LP) and secondary syphilis, particularly in immunocompromised patients. While LP is primarily an autoimmune condition, its presentation in this patient mimicked secondary syphilis, raising significant diagnostic concerns. This overlap highlights the need for clinicians to maintain a broad differential diagnosis when evaluating dermatological lesions, especially in populations with atypical presentations.

The immunocompromised state induced by chemotherapy likely contributed to the unusual manifestation of LP in this patient. It has been shown that drugs can induce lichen planus by acting as haptens that attach to keratinocytes or melanocytes, subsequently triggering a cytotoxic T lymphocyte response [15].

Histopathological evaluation was critical in this case, serving as the definitive diagnostic modality. The classic findings of hyperkeratosis, hypergranulosis, and a band-like lymphocytic infiltrate at the dermo-epidermal junction confirmed LP and ruled out other conditions, including secondary syphilis. Importantly, the histopathology of secondary syphilis can also exhibit a lichenoid pattern, creating significant overlap with LP. This emphasizes the necessity of utilizing special stains, such as those for *Treponema pallidum*, to confirm the diagnosis. Tissue biopsy becomes crucial when clinical and serological findings are inconclusive, particularly in patients with risk factors for multiple conditions.

The prognosis for LP is generally favorable and may resolve spontaneously within one to two years [16]. In this case, the combination of systemic apremilast and topical corticosteroids led to significant improvement within weeks, highlighting the efficacy of immunomodulatory therapy in managing LP. However, clinicians should be aware of potential relapses, especially in patients with ongoing immunosuppression, necessitating long-term follow-up.

For secondary syphilis, misdiagnosis can be severe, as untreated cases may progress to tertiary syphilis, causing significant morbidity and mortality from cardiovascular or central nervous system complications [17]. This reinforces the importance of accurate and timely diagnosis to guide treatment. While serologic testing is highly sensitive for syphilis, histopathology remains invaluable for excluding alternative diagnoses in ambiguous cases.

This case contributes to the growing awareness of atypical presentations of LP, particularly in immunocompromised patients. As LP can mimic infectious conditions like syphilis, it is imperative for clinicians to consider a range of potential diagnoses and employ a systematic approach to evaluation. Early recognition and management of LP not only improve patient outcomes but also prevent unnecessary treatments for misdiagnosed conditions, such as antibiotic therapy for presumed syphilis.

## Conclusions

Lichen planus is a chronic inflammatory condition that can sometimes mimic other dermatological diseases, such as secondary syphilis, presenting unique diagnostic challenges in patients. This case highlights the importance of histopathological confirmation in differentiating LP from infectious conditions such as syphilis, which require different management strategies. Clinicians should consider LP in the differential diagnosis of atypical palmoplantar and mucosal lesions. Early diagnosis and appropriate treatment, including topical and systemic therapies, can improve outcomes and prevent unnecessary interventions. Awareness of these overlapping presentations is essential for improving diagnostic accuracy and optimizing patient care.
